# The UNC-6/Netrin receptors UNC-40/DCC and UNC-5 inhibit growth cone filopodial protrusion via UNC-73/Trio, Rac-like GTPases and UNC-33/CRMP

**DOI:** 10.1242/dev.110437

**Published:** 2014-11-15

**Authors:** Adam D. Norris, Lakshmi Sundararajan, Dyan E. Morgan, Zachary J. Roberts, Erik A. Lundquist

**Affiliations:** Programs in Genetics and Molecular, Cellular, and Developmental Biology, Department of Molecular Biosciences, The University of Kansas, 1200 Sunnyside Avenue, Lawrence, KS 66045, USA

**Keywords:** UNC-40/DCC, UNC-5, UNC-6, Axon repulsion, Filopodia, Growth cone, *Caenorhabditis elegans*

## Abstract

UNC-6/Netrin is a conserved axon guidance cue that can mediate both attraction and repulsion. We previously discovered that attractive UNC-40/DCC receptor signaling stimulates growth cone filopodial protrusion and that repulsive UNC-40–UNC-5 heterodimers inhibit filopodial protrusion in *C. elegans*. Here, we identify cytoplasmic signaling molecules required for UNC-6-mediated inhibition of filopodial protrusion involved in axon repulsion. We show that the Rac-like GTPases CED-10 and MIG-2, the Rac GTP exchange factor UNC-73/Trio, UNC-44/Ankyrin and UNC-33/CRMP act in inhibitory UNC-6 signaling. These molecules were required for the normal limitation of filopodial protrusion in developing growth cones and for inhibition of growth cone filopodial protrusion caused by activated MYR::UNC-40 and MYR::UNC-5 receptor signaling. Epistasis studies using activated CED-10 and MIG-2 indicated that UNC-44 and UNC-33 act downstream of the Rac-like GTPases in filopodial inhibition. UNC-73, UNC-33 and UNC-44 did not affect the accumulation of full-length UNC-5::GFP and UNC-40::GFP in growth cones, consistent with a model in which UNC-73, UNC-33 and UNC-44 influence cytoskeletal function during growth cone filopodial inhibition.

## INTRODUCTION

**Fig. 1.**
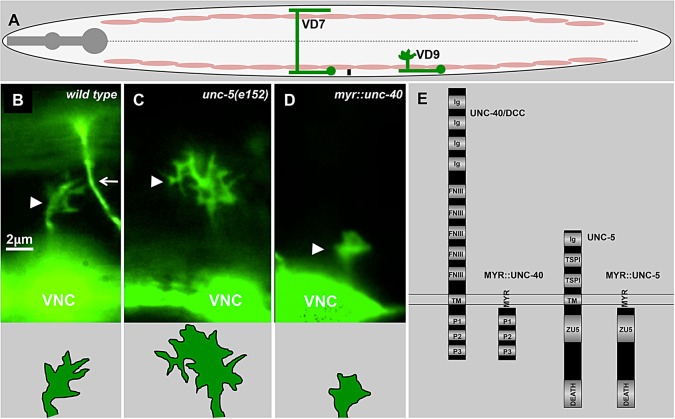
**UNC-40 and UNC-5 signaling inhibits growth cone protrusion.** (A) Diagram of an L2 hermaphrodite *C. elegans* highlighting the position and structure of the VD neurons. Anterior is to the left, and dorsal is up. The pink ovals represent the ventral and dorsal muscle quadrants, and the dashed line indicates the lateral midline. The cell bodies of the 13 VD neurons reside along the ventral nerve cord (VNC). In the early L2 larval stage, they extend axons anteriorly in the VNC. The axons then turn dorsally and migrate to the dorsal nerve cord to form commissures. Only two of the 13 VD neurons are shown (green). While migrating between the ventral muscle quadrant and the lateral midline, the VD growth cones display an expanded, protrusive morphology with multiple dynamic filopodial protrusions. VD7 shows the final structure of the VD neurite, and VD9 shows a dorsally directed commissural process with a growth cone that is migrating between the ventral muscle quadrant and lateral midline, which is the position at which VD growth cone morphology is analyzed in this work. (B-D) Fluorescence micrographs of early L2 animals with GFP expression in the VD growth cones (*juIs76[Punc-25::gfp]*) (arrowheads). The bottom panels are tracings of the VD growth cones above. The arrow (B) points to a DD commissural axon that extended earlier in development. Compared with wild type, the *unc-5(e152)* growth cone is larger and more protrusive, with longer and more persistent filopodia that are not all directed to the dorsal side as in wild type. By contrast, activated *myr::unc-40* growth cones (*lqIs128[myr::unc-40]*) are smaller and less protrusive. Dorsal is up, and anterior is left. For a detailed description of growth cone behaviors, see Norris and Lundquist (2011). (E) Diagram of the UNC-40 and UNC-5 guidance receptors, and the myristoylated versions used in this work. Ig, immunoglobulin-like domain; FNIII, Fibronectin type III domain; TM, transmembrane domain; P1-3, proline-rich domains; MYR, myristoylation signal; TSP1, Thrombospondin type I domain.

Extracellular guidance cues are detected by receptors on the growth cone and guide growth cone migration. The guidance cue UNC-6/Netrin and its receptors UNC-5 and UNC-40/DCC control both attraction and repulsion in the dorsal-ventral axis (Chan et al., 1996; Leonardo et al., 1997; Hong et al., 1999; Montell, 1999; Shekarabi and Kennedy, 2002; Moore et al., 2007). UNC-40/DCC homodimers mediate attraction to Netrin, and UNC-5–UNC-40 heterodimers mediate repulsion from Netrin (Hong et al., 1999; MacNeil et al., 2009). In *C. elegans*, UNC-6, UNC-40 and UNC-5 mediate the dorsal-ventral circumferential migrations of growth cones and their axons (Hedgecock et al., 1990; Ishii et al., 1992; Leung-Hagesteijn, 1992; Chan et al., 1996). The VD motor neurons extend axons dorsally in a circumferential manner (Fig. 1A) and are repelled from a ventral source of UNC-6 (Hedgecock et al., 1990; Norris and Lundquist, 2011). As growth cones migrate, they extend dynamic lamellipodial and filopodial protrusions in the direction of migration. The repelled VD growth cones display dorsally directed dynamic lamellipodial and filopodial protrusions, with fewer protrusions directed ventrally (Fig. 1B) (Knobel et al., 1999; Norris and Lundquist, 2011). The roles of UNC-6/Netrin and its receptors in attractive and repulsive axon guidance are well documented. However, less is known about cell biological mechanisms of axon guidance and the regulation of growth cone protrusion by axon guidance signaling pathways such as UNC-6/Netrin.

Previously, we discovered a link between axon guidance and the regulation of growth cone protrusion by UNC-6, UNC-40 and UNC-5 (Norris and Lundquist, 2011). Genes involved in the attraction to UNC-6 were required for growth cone protrusion, including filopodia, and those involved in repulsion were required to inhibit growth cone filopodial protrusion (Norris and Lundquist, 2011). VD growth cones, repelled from UNC-6, are highly dynamic and display dorsally directed filopodial protrusions with an average maximal length of 1 µm and average duration of 5 min (Norris and Lundquist, 2011) (Fig. 1A,B; supplementary material Movie 1). Loss of UNC-6 and UNC-5 resulted in VD growth cones that were larger and more protrusive (Fig. 1C; supplementary material Movie 2), and activation of UNC-5 and UNC-40 using a myristoylated version of the UNC-40 cytoplasmic domain (Gitai et al., 2003) resulted in small growth cones with very little filopodial protrusion (Norris and Lundquist 2011) (Fig. 1D,E; supplementary material Movie 3). UNC-40 was required for the excess protrusion in *unc-5* mutants, indicating that, in repelled VD growth cones, UNC-6 and UNC-40 control both pro- and anti-protrusive activity. Furthermore, UNC-6 and UNC-5 were also required to bias protrusion asymmetrically to the dorsal side of the growth cone (i.e. in *unc-5* mutants protrusions were observed both dorsally and ventrally as opposed to mainly dorsally in wild type) (Fig. 1B,C) (Norris and Lundquist, 2011). These data suggest a mechanism of axon repulsion by a balance of UNC-6-mediated pro- and anti-protrusive forces in the growth cone, with pro-protrusive forces (UNC-40 homodimers) predominating dorsally, distant from the UNC-6 source, and anti-protrusive forces (UNC-5–UNC-40 heterodimers) predominating ventrally, adjacent to the UNC-6 source.

Signaling pathways required for UNC-40/DCC-mediated attractive axon guidance have been extensively described (reviewed by Lai Wing Sun et al., 2011). In *C. elegans*, these pathways drive neuronal lamellipodial and filopodial protrusion. For example, the Rac-like GTPases CED-10 and MIG-2, CDC-42, UNC-34/Enabled, the Arp2/3 complex, the Rac-specific guanine nucleotide exchange factor (GEF) TIAM-1, and the actin-interacting protein UNC-115/abLIM stimulate neuronal protrusion and mediate attractive axon guidance in response to UNC-40 (Gitai et al., 2003; Struckhoff and Lundquist, 2003; Shakir et al., 2008; Norris et al., 2009; Demarco et al., 2012; Alan et al., 2013). The Arp2/3 complex, UNC-34 and UNC-115 are required for growth cone filopodia formation (Norris et al., 2009). However, the roles of these molecules differ in different growth cones. For example, in the longitudinally migrating PLM touch sensory growth cones, VAB-1/EphR signaling inhibits growth cone filopodia and outgrowth by activating Arp2/3 and inhibiting UNC-34 (Mohamed et al., 2012).

Less is known about mechanisms of UNC-6/Netrin-based repulsion, although Src and Fak kinases, the PAK-like molecule MAX-2, the PH/MyTH4/FERM adaptor protein MAX-1, and the SHP2 tyrosine phosphatase are important (Tong et al., 2001; Huang et al., 2002; Killeen et al., 2002; Li et al., 2006; Lucanic et al., 2006). In this work we use the activated MYR::UNC-40-encoding transgene expressed in repelled VD neurons to decipher mechanisms of growth cone filopodial inhibition by UNC-6 receptor signaling in repulsive axon guidance.

Rac GTPases and Trio GEFs have central roles in axon guidance (Steven et al., 1998; Bateman et al., 2000; Blangy et al., 2000; Lundquist et al., 2001; Kishore and Sundaram, 2002; Lundquist, 2006). UNC-73/Trio acts as a GEF for Rac GTPases and is required for proper neuronal migration and axon guidance, including the VD commissural axons. In *Drosophila* and vertebrates, Trio interacts with the Netrin receptor DCC and activates Rac in response to Netrin (Forsthoefel et al., 2005; Briancon-Marjollet et al., 2008; DeGeer et al., 2013). In these cases, Trio apparently acts in attractive axon guidance mediated by Netrin, suggesting that it might stimulate protrusion. However, in *C. elegans*, *unc-73* is not required for UNC-40-stimulated neuronal protrusion (Gitai et al., 2003), which requires the Rac GEF TIAM-1 (Demarco et al., 2012). Here, we show that the Rac-like GTPases CED-10 and MIG-2 and UNC-73 inhibit growth cone filopodial protrusion. Our results suggest that CED-10 and MIG-2 are involved in both pro- and anti-protrusive functions in the growth cone and that their roles in each are controlled by distinct GEFs: UNC-73 in inhibition (this work) and TIAM-1 in stimulation (Demarco et al., 2012) of protrusion.

*unc-33* encodes a protein that is similar to the Collapsin response mediator protein (CRMP) (Li et al., 1992), which mediates growth cone collapse in response to the Semaphorin/Collapsin family of repulsive axon guidance cues (Goshima et al., 1995; Takahashi et al., 1999; Alabed et al., 2007, 2010). *unc-33* is required for axon guidance and for regulating axonal versus dendritic sorting of trafficked molecules (Li et al., 1992; Maniar et al., 2012). *unc-44* encodes an Ankyrin-like molecule that is involved in the axonal localization of UNC-33 (Otsuka et al., 1995; Maniar et al., 2012). Here, we show that UNC-33/CRMP and UNC-44/Ankyrin are required by MYR::UNC-40 to limit growth cone filopodial protrusion, and that they act downstream of Rac GTPases in this process.

Previous studies found that UNC-73/Trio and MIG-2/RhoG affected the accumulation and distribution of the SAX-3/Robo and UNC-40 receptors in neurons (Levy-Strumpf and Culotti, 2007; Watari-Goshima et al., 2007), and that UNC-33 and UNC-44 affect axon-dendrite trafficking (Maniar et al., 2012). We show that full-length UNC-40::GFP and UNC-5::GFP localization to growth cones is unaffected by *unc-73*, *unc-44* and *unc-33*, consistent with these molecules acting downstream of UNC-6 receptor signaling.

## RESULTS

### The Rac GEF UNC-73 is required to inhibit VD growth cone filopodial protrusion

**Fig. 2.**
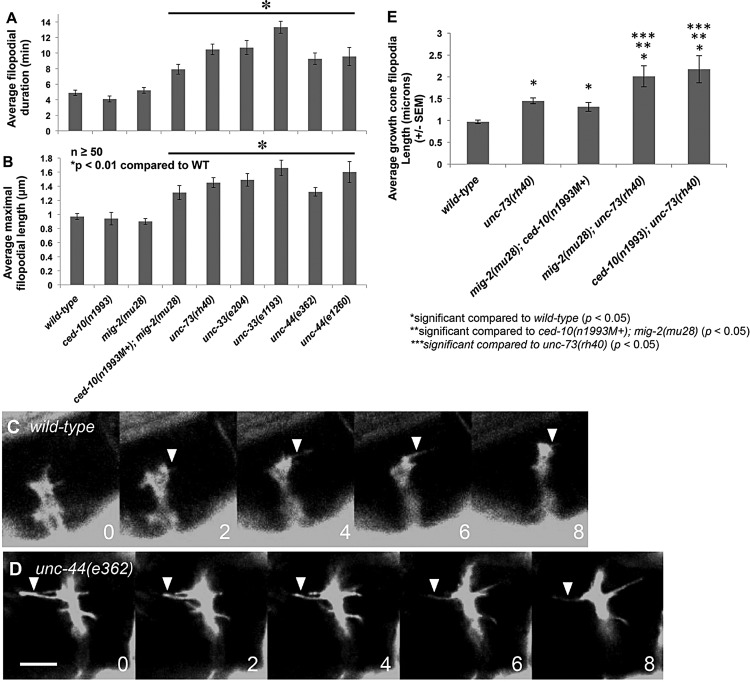
**Mutations in Rac GTPases, UNC-73, UNC-44 and UNC-33 cause increased growth cone filopodial protrusion.** (A) The average VD growth cone filopodial duration in different mutant backgrounds in early L2 *juIs76* animals. More than 50 filopodia per genotype were scored. (B) Maximal filopodial length in different mutants as described in A. (C) A time-lapse series of a wild-type VD growth cone in early L2. Numbers indicate minutes after imaging began. (D) An *unc-44(e362)* mutant growth cone showing increased protrusion in the form of longer and more persistent filopodia. Dorsal is up, and anterior is left. Scale bar: 5 µm. (E) Average filopodial length in different genotypes. At least 15 filopodia were scored from at least 15 different growth cones. M+ indicates that the animals had wild-type maternal *ced-10(+)* activity. (A,B,E) Error bars represent s.e.m. Two-sided *t-*tests with unequal variance were used to determine statistical significance.

**Fig. 3.**
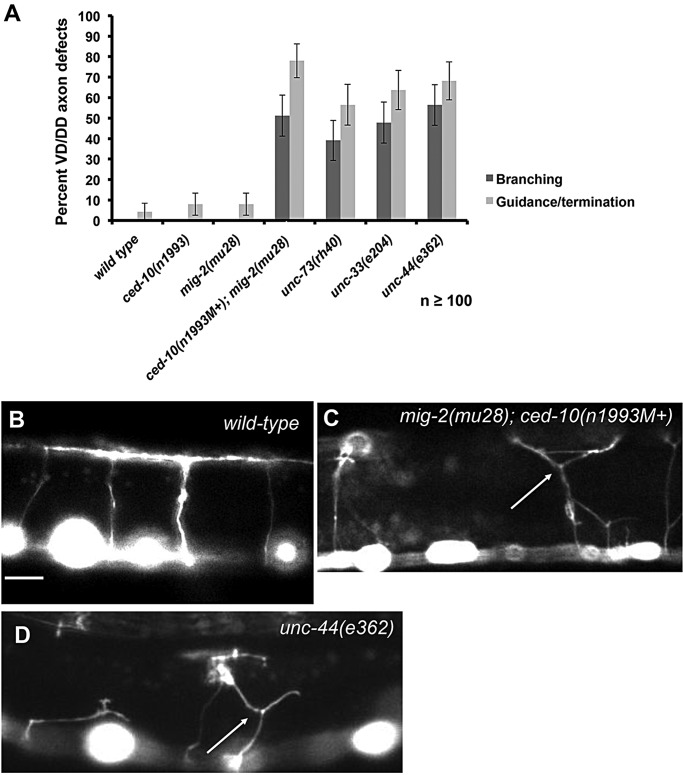
**Mutations with increased growth cone protrusion cause ectopic axon branching.** (A) Quantification of VD/DD axon pathfinding defects (guidance and premature termination) and ectopic axon branching in young adults. At least 100 axons were scored. Error bars represent 2× standard error of proportion. (B-D) Representative fluorescence micrographs of VD/DD axons demonstrating axon pathfinding and branching errors. (B) Wild-type, (C) *mig-2(mu28); ced-10(n1993M+)* and (D) *unc-44(e362)* VD/DD axons. Arrows point to ectopic axon branches. Dorsal is up, and anterior is left. M+ indicates that the animals had wild-type maternal *ced-10(+)* activity. Scale bar: 10 µm.

The *unc-73(rh40)* mutation eliminates the Rac GEF activity of UNC-73 but does not affect other activities (e.g. Rho GEF activity) (Steven et al., 1998; Lundquist et al., 2001; Demarco et al., 2012). We found that in *unc-73(rh40)* mutants VD growth cones had significant increases in filopodial protrusiveness, exhibiting on average longer filopodia (e.g. 0.96 µm in wild type compared with 1.44 µm in *unc-73(rh40)*; *P<*0.01) and a longer duration of the filopodia once formed (4.9 min in wild type compared with 8.2 min in *unc-73(rh40)*; *P*<0.01) (Fig. 2A,B; supplementary material Movie 4). Indeed, some filopodia endured throughout the length of the experiment (greater than 20 min). In some cases, the exceptionally long filopodia consolidated into neurites, resulting in a terminated axon with extensive branching. Indeed, *unc-73* mutant adults exhibit extensive branching of the PDE neurons (Struckhoff and Lundquist, 2003) and of the VD and DD axons (Fig. 3), suggesting that failure to retract growth cone filopodia can result in the formation of ectopic neurites and axon branching. *unc-5* mutants also displayed persistent growth cone filopodial extensions as well as axon branching (Norris and Lundquist, 2011).

### The Rac-like GTPases CED-10 and MIG-2 inhibit growth cone filopodial protrusion

CED-10 is similar to the Rho GTPase Rac1 (Reddien and Horvitz, 2000) and MIG-2 is an Mtl GTPase (Zipkin et al., 1997), which is an invertebrate-specific Rho GTPase family with similarity to both Rac and Cdc42. MIG-2 might be the functional equivalent of the vertebrate GTPase RhoG (deBakker et al., 2004). We refer to MIG-2 and CED-10 collectively as the *C. elegans* Rac GTPases. CED-10/Rac and MIG-2/RhoG redundantly control axon guidance and mediate neuronal protrusion downstream of UNC-40/DCC in neurons with axons attracted to UNC-6/Netrin (Struckhoff and Lundquist, 2003; Demarco et al., 2012). However, *ced-10; mig-2* double mutants also exhibit extensive axon branching in the PDE and VD/DD neurons (Fig. 3) (Lundquist et al., 2001; Struckhoff and Lundquist, 2003), suggesting possible involvement in inhibition of filopodial protrusion. *ced-10* and *mig-2* single mutants showed no significant defects in VD growth cone filopodial length or duration (Fig. 2A). *mig-2; ced-10* double-mutant VD growth cones exhibited increased filopodial length and persistence similar to *unc-73* (Fig. 2; supplementary material Movie 5), indicating that CED-10 and MIG-2 act redundantly in limiting filopodial length and duration in repelled VD growth cones.

The Rac GEF domain of UNC-73/Trio acts as a GEF on CED-10/Rac and MIG-2/RhoG, but not *C. elegans* CDC-42 (Wu et al., 2002). To explore the interaction of CED-10, MIG-2 and UNC-73 further, we analyzed average filopodial length in double mutants. *mig-2(mu28); unc-73(rh40)* and *ced-10(n1993); unc-73(rh40)* displayed increased average growth cone filopodial length compared with wild type, the *ced-10; mig-2* double mutant, and *unc-73(rh40)* (Fig. 2E). That *unc-73(rh40)* is null for Rac GEF activity (Steven et al., 1998) suggests that another Rac GEF acts in parallel to UNC-73. *ced-10(n1993)* is not a null mutation and retains some function (Reddien and Horvitz, 2000; Shakir et al., 2006), and *mig-2(mu28); ced-10(n1993)* mutants have wild-type *ced-10(+)* maternal contribution, so this double mutant does not represent a complete loss of *ced-10* and *mig-2* function, which might have a more severe filopodial phenotype than *mig-2(mu28); ced-10(n1993)*.

### UNC-44 and UNC-33 inhibit VD growth cone filopodial protrusion

*unc-33* and *unc-44* mutants display branched and prematurely terminated axons, including the VDs (Fig. 3), indicative of a role in filopodial inhibition. VD growth cone time-lapse analysis indicated that *unc-33* and *unc-44* mutant growth cones display excessive filopodial protrusion similar to *unc-73*, *mig-2; ced-10*, and *unc-5* (e.g. increased filopodial length and duration) (Fig. 2; supplementary material Movies 6 and 7). *unc-33* and *unc-44* also displayed persistent filopodia that resolved into stable neurite-like structures (supplementary material Movies 6 and 7), resulting in axon branching (Fig. 3).

### UNC-73, UNC-33 and UNC-44 are required for activated MYR::UNC-40 inhibition of VD growth cone protrusion

**Fig. 4.**
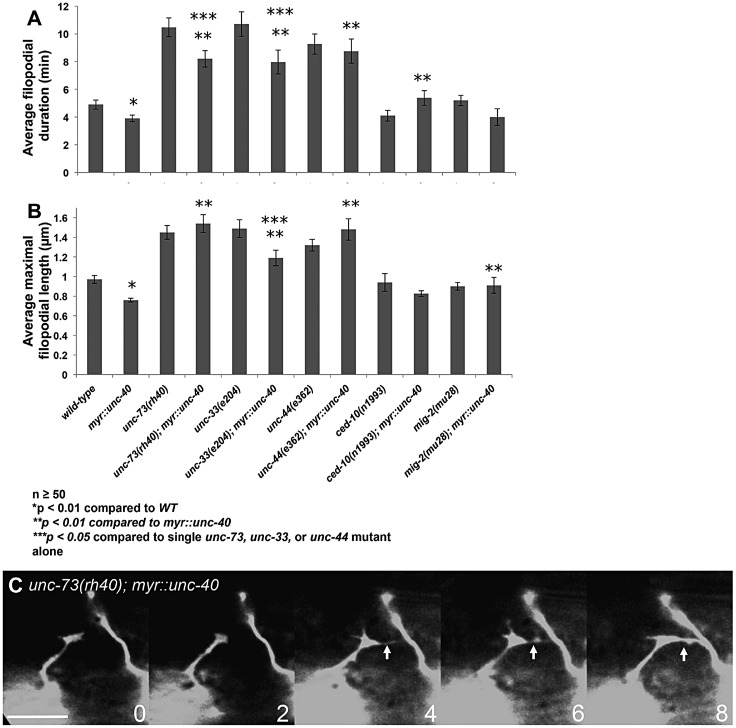
**UNC-73, UNC-33 and UNC-44 are required for *myr::unc-40* filopodial inhibition.** (A,B) Quantification of filopodia dynamics in VD growth cones as described in Fig. 2A. Error bars represent s.e.m. Two-sided *t-*tests with unequal variance were used to determine statistical significance. (C) Time-lapse series of an *unc-73(rh40); myr::unc-40* growth cone, taken at 2 min per frame. The arrow points to a long, stable filopodium of a type that was never observed in *myr::unc-40* alone but often observed in *unc-73(rh40)*. Dorsal is up, and anterior is left. Scale bar: 5 µm.

We next determined whether UNC-73, UNC-33 and UNC-44 are required for filopodial inhibition driven by activated MYR::UNC-40 (see Fig. 1). VD growth cones in loss-of-function mutants of *unc-73*, *unc-33* and *unc-44* harboring *myr::unc-40* resembled the loss-of-function mutants alone (i.e. increased growth cone filopodial protrusiveness as indicated by length and duration) (Fig. 4; supplementary material Movie 8). Additional alleles *unc-33(e1197)* and *unc-44(e1193)* showed the same effect (data not shown). Thus, UNC-73, UNC-33 and UNC-44 are required for inhibition of growth cone protrusion mediated by MYR::UNC-40. These results are in line with previous studies that identified *unc-44* mutations in a screen for suppressors of UNC-5 axon repulsion activity (Colavita and Culotti, 1998).

*ced-10(n1993)* weakly but significantly suppressed the filopodial duration of *myr::unc-40*, and *mig-2(mu28)* weakly but significantly suppressed maximal length (Fig. 4A,B). This weak suppression is likely to be due to the demonstrated redundancy of MIG-2 and CED-10 in filopodial inhibition.

While *unc-73*, *unc-33* and *unc-44* were epistatic to *myr::unc-40*, the duration of filopodia in *unc-33(e204); myr::unc-40* and *unc-73(rh40); myr::unc-40* was significantly lower than that of *unc-33(e204)* and *unc-73(rh40)* alone, and the maximal length of *unc-33; myr::unc-40* was significantly reduced compared with *unc-33(e204)* alone. This suggests that *myr::unc-40* can slightly suppress *unc-33* and *unc-73*, indicating that it might engage effectors in parallel to *unc-33* and *unc-73* to inhibit protrusion.

### CED-10 and MIG-2 require UNC-33 and UNC-44 to limit growth cone filopodial protrusion

**Fig. 5.**
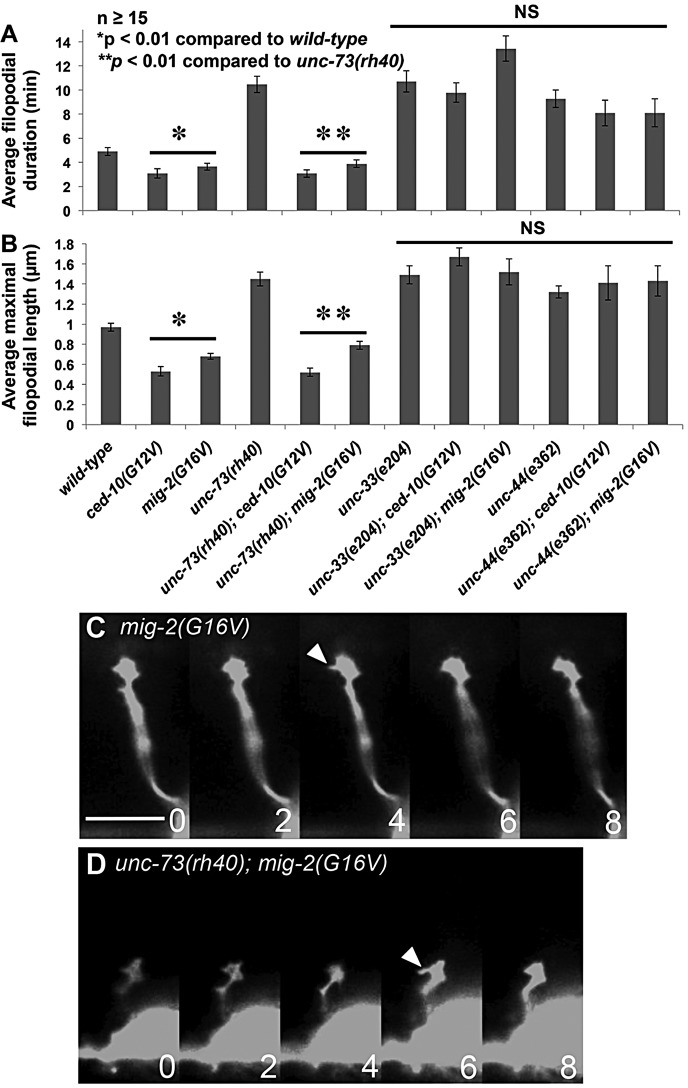
**UNC-33 and UNC-44, but not UNC-73, are required for constitutively active MIG-2 and CED-10 filopodial inhibition.** (A,B) Quantification of filopodia dynamics in VD growth cones as described in Fig. 2A. Error bars represent s.e.m. Two-sided *t-*tests with unequal variance were used to determine statistical significance. NS, not significant. (C,D) Time-lapse series of live growth cones (arrowheads) in early L2 animals, taken at 2 min per frame. Dorsal is up, and anterior is left. The *unc-73(rh40); mig-2(G16V)* growth cone resembled that of *mig-2(G16V)* alone and did not exhibit the excess protrusion seen in *unc-73(rh40)* mutants. Scale bar: 5 µm.

Our results indicate that the Rac GTPases CED-10 and MIG-2 are required to limit VD growth cone filopodial protrusion. We generated activated *ced-10(G12V)* and *mig-2(G16V)* expressed in the VD neurons by the *unc-25* promoter (see Materials and Methods). In the PDE axons that are attracted to UNC-6, activated CED-10(G12V) and MIG-2(G16V) result in excess protrusion (Struckhoff and Lundquist, 2003). However, in the repelled VD axons, CED-10(G12V) and MIG-2(G16V) resulted in growth cones that displayed reduced filopodial protrusion compared with wild type (Fig. 5; supplementary material Movie 9), with a reduction in average filopodia duration [e.g. 4.9 min in wild type compared with 3.6 min in *mig-2(G16V)*; *P*<0.01] and length [0.96 µm in wild type compared with 0.68 µm in *mig-2(G16V)*; *P*<0.01]. This phenotype is the opposite of that observed in *unc-73* and *mig-2; ced-10* loss-of-function mutants, and resembled inhibition of growth cone protrusion caused by activated *myr::unc-40* (Norris and Lundquist, 2011). CED-10 and MIG-2 have pro-protrusive roles in other neurons with axons attracted to UNC-6 (Struckhoff and Lundquist, 2003; Demarco et al., 2012). In the VD growth cones repelled from UNC-6, MIG-2 and CED-10 have an anti-protrusive role.

When *ced-10(G12V)* and *mig-2(G16V)* were introduced into an *unc-73(rh40)* loss-of-function background, the growth cones resembled those upon activated Rac GTPase expression alone, including significant reduction in filopodia duration and length (Fig. 5; supplementary material Movie 10). That activated CED-10 and MIG-2 were epistatic to *unc-73* could mean that CED-10 and MIG-2 act downstream of UNC-73 in the same pathway, or that they act independently of UNC-73 in a parallel pathway, consistent with the double-mutant analysis in Fig. 2E.

By contrast, double mutants of *unc-33* and *unc-44* with *ced-10(G12V)* and *mig-2(G16V)* resembled *unc-33* and *unc-44* mutants (*unc-33* and *unc-44* were epistatic to activated Rac GTPases), with excessive growth cone filopodial protrusion as evidenced by increased filopodial length and duration (Fig. 5). Additional alleles *unc-33(e1197)* and *unc-44(e1193)* showed the same effect (data not shown). These data indicate that UNC-33 and UNC-44 are required for Rac GTPases to inhibit growth cone protrusion and suggest that they act downstream of Rac GTPases in the process.

### Mutants with increased filopodial protrusion exhibit increased axon branches

**Fig. 6.**
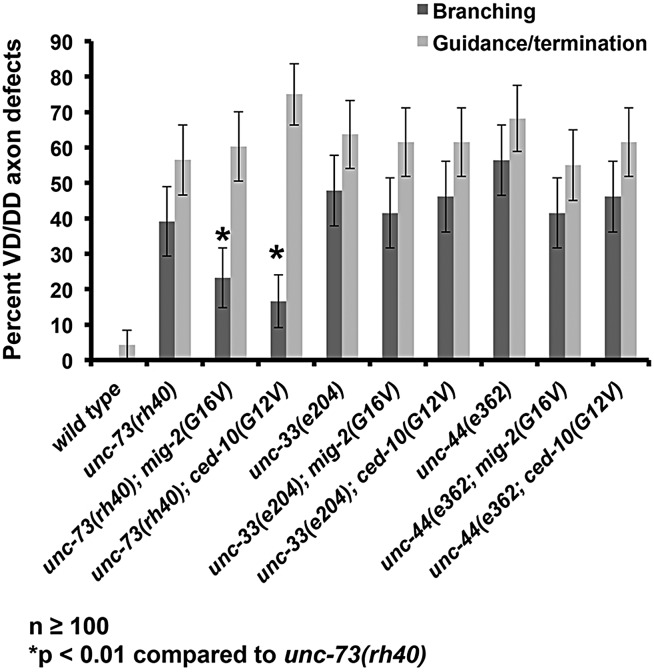
**Excessive filopodial protrusion correlates with axon branching defects in adult animals.** Quantification of VD/DD axon pathfinding defects and ectopic axon branches as described in Fig. 3. Activated *ced-10(G12V)* and *mig-2(G16V)*, which suppress excessive growth cone filopodial protrusion of *unc-73(rh40)*, also suppress ectopic axon branching but not other guidance defects. *ced-10(G12V)* and *mig-2(G16V)* did not suppress excessive growth cone filopodial protrusion of *unc-33* and *unc-44*, nor did they suppress ectopic axon branching of these mutants. M+ indicates that the animals had wild-type maternal *ced-10(+)* contribution. Error bars represent 2× standard error of proportion. Fisher's exact tests were used to determine statistical significance.

Our results suggest a correlation between increased growth cone filopodial protrusion and ectopic axon branches in the adult animal (Fig. 3). The stable and long filopodia in mutant growth cones might be the precursors to these ectopic axon branches. To test this idea, we studied the effects of activated Rac GTPases on ectopic axon branching. Activated Rac GTPases suppressed excess growth cone filopodial protrusion in *unc-73* but not *unc-33* and *unc-44* (Fig. 5). In adults, *unc-73(rh40); mig-2(G16V)* and *unc-73(rh40); ced-10(G12V)* mutants displayed reduced ectopic VD axon branching compared with *unc-73(rh40)* alone (Fig. 6), similar to the effect on growth cone filopodial protrusion. This effect was specific to axon branching defects, as overall pathfinding defects (axons that wander, or that stop short of the dorsal nerve cord) remained unchanged. By contrast, *mig-2(G16V)* or *ced-10(G12V)* did not reduce ectopic axon branches of *unc-33* and *unc-44* mutants (Fig. 6), which is also similar to their effects on growth cone filopodial protrusion. These data support the idea that axon branches in adult axons can result from failure to inhibit the extent of filopodia protrusion in the developing growth cone.

### MYR::UNC-5 inhibits growth cone protrusion in a manner dependent on UNC-73 and UNC-33

**Fig. 7.**
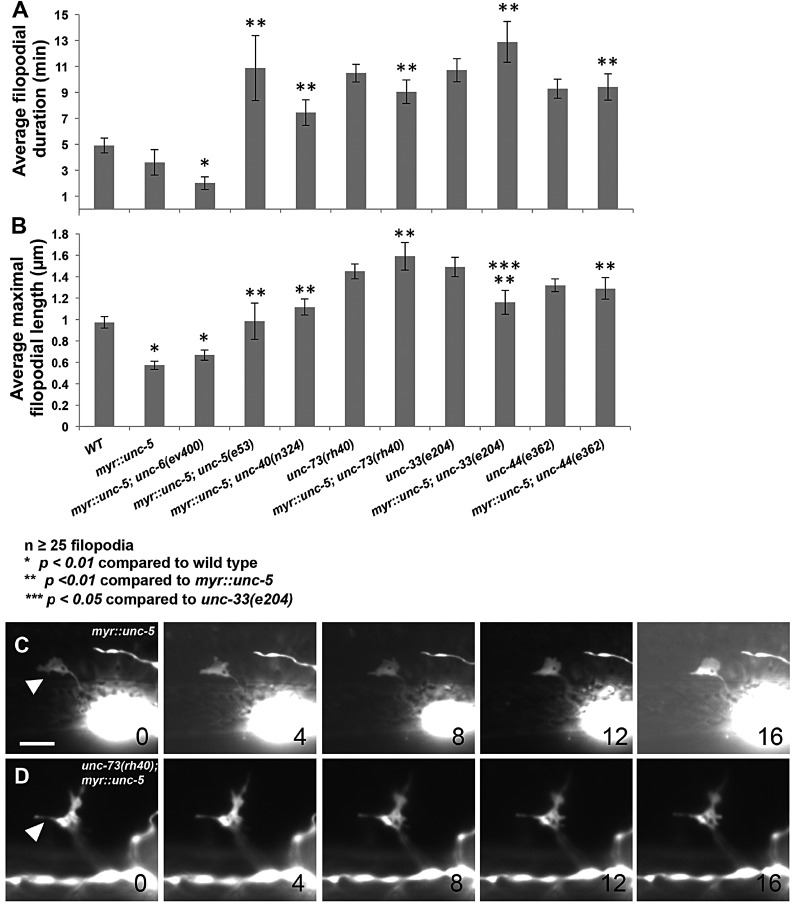
**MYR::UNC-5 inhibits growth cone filopodial protrusion similar to MYR::UNC-40.** (A,B) Quantification of filopodia dynamics in VD growth cones as described in Fig. 2A. *myr::unc-5* indicates a transgene that drives *myr::unc-5* expression from the *unc-25* promoter (see Materials and Methods and Fig. 1A). Two-sided *t-*tests with unequal variance were used to determine statistical significance. (C) Time-lapse series of a *myr::unc-5* growth cone in an early L2 animal, taken at 4 min per frame. The arrowhead points to a growth cone with limited protrusion. (D) Time-lapse series of an *unc-73(rh40); myr::unc-5* growth cone with long and persistent protrusions (arrowhead). Dorsal is up, and anterior is left. Scale bar: 5 µm.

Functional UNC-5 was required for the anti-protrusive effects of MYR::UNC-40, suggesting that an UNC-5–MYR::UNC-40 complex is involved in inhibiting growth cone protrusion (Norris and Lundquist, 2011). To test the effects of activated UNC-5 signaling, we constructed a transgene with a myristoylated version of the UNC-5 cytoplasmic domain (MYR::UNC-5) expressed in the VD neurons using the *unc-25* promoter (Fig. 1E). Expression of MYR::UNC-5 caused reduced VD growth cone filopodial protrusion that resembled the effect of MYR::UNC-40, including decreased filopodial length and duration (Fig. 7A-C; supplementary material Movie 11). This effect was not dependent upon functional UNC-6, but was dependent upon functional UNC-40, suggesting that a MYR::UNC-5–UNC-40 heterodimeric complex was involved (Fig. 7A,B). Surprisingly, functional endogenous UNC-5 was also required (Fig. 7A,B). Functional endogenous UNC-40 was not required for the effect of MYR::UNC-40 (Norris and Lundquist, 2011). This result suggests the involvement of UNC-5–UNC-40 heterodimers as well as UNC-5 homodimers that require at least one full-length UNC-5 molecule. *unc-73(rh40)*, *unc-33* and *unc-44* loss-of-function mutations suppressed the anti-protrusive activity of *myr::unc-5* (Fig. 7A,B), consistent with a model in which MYR::UNC-5 inhibits protrusion using UNC-73 Rac GEF activity and UNC-33, similar to MYR::UNC-40. *myr::unc-5* reduced the maximal filopodial length of *unc-33(e204)* (Fig. 7B), indicating that MYR::UNC-5 might engage effectors in parallel to UNC-33 to inhibit protrusion.

### UNC-40::GFP and UNC-5::GFP accumulation in growth cones is unaffected by *unc-73*, *unc-33* and *unc-44*

**Fig. 8.**
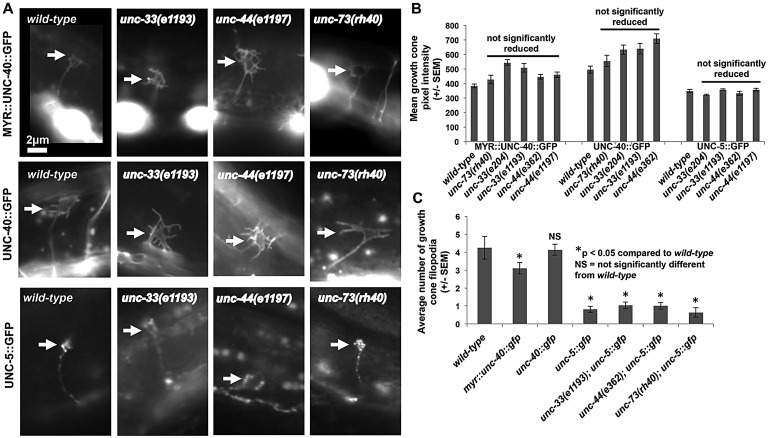
***unc-73*, *unc-33* and *unc-44* do not affect UNC-40::GFP or UNC-5::GFP accumulation in growth cones.** (A) Fluorescence micrographs of MYR::UNC-40::GFP, full-length UNC-40::GFP and full-length UNC-5::GFP accumulation in VD growth cones in the indicated mutant backgrounds. Arrows point to growth cones. (B) Quantification of the mean pixel intensity of MYR::UNC-40::GFP, full-length UNC-40::GFP and full-length UNC-5::GFP in VD growth cones (see Materials and Methods). At least ten growth cones for each genotype were analyzed, except for *unc-73(rh40); unc-5::gfp*, which were subviable and sterile. *unc-73(rh40); unc-5::gfp* growth cones were not quantified, but those observed showed no gross change in UNC-5::GFP growth cone localization. (C) The average number of growth cone filopodia in different backgrounds (see Materials and Methods). At least ten growth cones were analyzed for each genotype. (B,C) Error bars represent s.e.m. Two-sided *t-*tests with unequal variance were used to determine statistical significance.

In wild type, MYR::UNC-40::GFP accumulated uniformly at the edges of the VD growth cones. Although MYR::UNC-40 is likely to be trafficked to the growth cone by a mechanism distinct from endogenous UNC-5 and UNC-40, its retention or stability in the growth cone could depend upon endogenous UNC-5. MYR::UNC-40::GFP displayed a grossly similar growth cone accumulation in *unc-73*, *unc-33* and *unc-44* mutants (Fig. 8A,B) despite increased growth cone protrusion. Indeed, levels of MYR::UNC-40::GFP were often increased in these mutants (Fig. 8B), possibly owing to their increased growth cone size and protrusions.

We constructed a full-length *unc-40::gfp* transgene (Levy-Strumpf and Culotti, 2007; Sundararajan and Lundquist, 2012) driven by the *unc-25* promoter in the VD neurons. Full-length UNC-40::GFP accumulated at the edges of the VD growth cones (Fig. 8A) and caused no apparent defects in growth cone morphology, as determined by number of filopodial protrusions on growth cones (Fig. 8C). UNC-40::GFP localization was grossly similar in *unc-73*, *unc-33* and *unc-44* mutants (Fig. 8A,B) and levels were increased in some cases, probably owing to increased growth cone protrusion and size in these mutants.

We used a full-length functional UNC-5::GFP-encoding transgene (Killeen et al., 2002) to test whether full-length UNC-5::GFP growth cone accumulation was affected. Full-length UNC-5::GFP accumulated in puncta that were located in the growth cone body and at the growth cone edges, as well as in the axon (Fig. 8A). A grossly similar distribution of UNC-5::GFP was observed in growth cones and axons in *unc-33(e1193)*, *unc-44(e1197)* and *unc-73* mutants (Fig. 8). *unc-33(e204)* and *unc-44(e362)* alleles were also analyzed with similar results (data not shown).

Wild-type growth cones expressing full-length UNC-5::GFP were small and had significantly fewer filopodial protrusions compared with wild type (Fig. 8A,C), suggesting that full-length UNC-5 transgenic expression can inhibit filopodial protrusion. In *unc-33*, *unc-44* and *unc-73* mutants, the growth cones were still small and displayed significantly reduced numbers of filopodial protrusions that were the same as those associated with full-length UNC-5::GFP alone (Fig. 8C). This is contrast to MYR::UNC-5 and MYR::UNC-40, which were suppressed by *unc-73*, *unc-44* and *unc-33*. Possibly, transgenic expression of full-length UNC-5::GFP has a stronger gain-of-function effect than MYR::UNC-5 that cannot be overcome by loss of UNC-73, UNC-33 and UNC-44. This also suggests the possibility of redundant downstream mechanisms in growth cone inhibition by UNC-5, i.e. full-length UNC-5::GFP might engage multiple downstream mechanisms more robustly than MYR::UNC-5, and loss of one pathway does suppress this effect.

These data indicate that localization of functional UNC-40::GFP and UNC-5::GFP to growth cones is grossly normal in *unc-73*, *unc-33* and *unc-44* mutants, suggesting that these molecules are likely to act downstream of UNC-40 and UNC-5. By contrast, a previous study in *C. elegans* sensory neurons described evidence that UNC-73 can also act upstream to alter trafficking of UNC-40::GFP (Levy-Strumpf and Culotti, 2007). Although UNC-73 could potentially exert a similar effect in VD growth cones that our assays did not detect, the results reported here are consistent with the idea that UNC-73, UNC-33 and UNC-44 do not affect UNC-5 and UNC-40 accumulation in VD growth cones but rather act downstream to mediate changes in growth cone protrusion.

## DISCUSSION

**Fig. 9.**
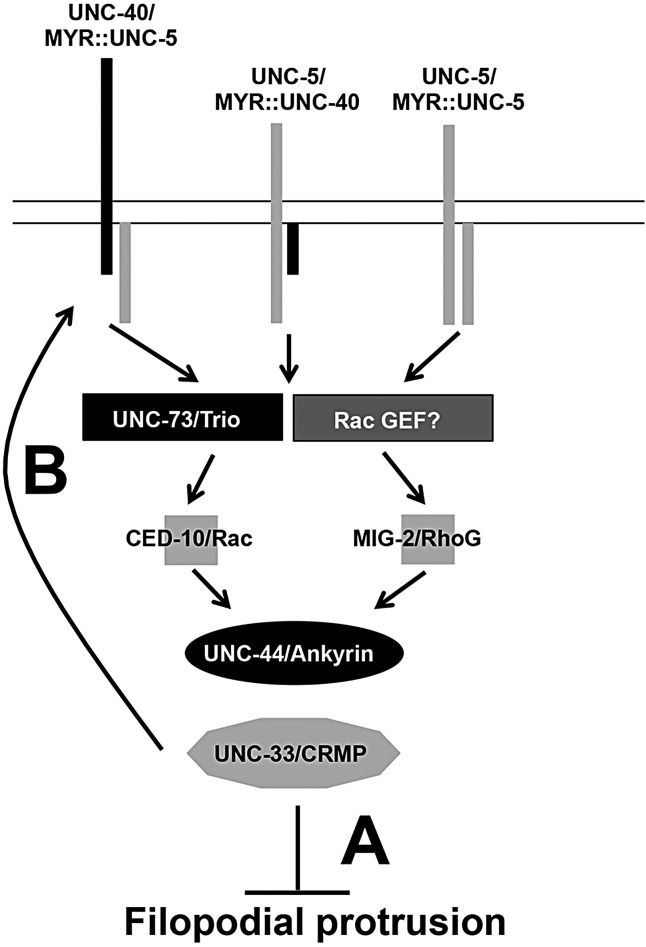
**MYR::UNC-5 and MYR::UNC-40 inhibit growth cone protrusion via UNC-73/Trio, Rac GTPases, UNC-44/Ankyrin and UNC-33/CRMP.** Data presented here and by Norris and Lundquist (2011) indicate that different receptor configurations inhibit growth cone protrusion, including MYR::UNC-40–UNC-5, MYR::UNC-5–UNC-40 and MYR::UNC-5–UNC-5. These complexes require the Rac GEF UNC-73/Trio, the Rac GTPases MIG-2/RhoG and CED-10/Rac, and the cytoskeleton-associated molecules UNC-44/Ankyrin and UNC-33/CRMP. The data presented here favor a model whereby UNC-73/Trio, Rac GTPases, UNC-44/Ankyrin and UNC-33/CRMP act downstream of the receptors, possibly modifying cytoskeletal dynamics (A). It is also possible that these molecules are required for growth cone localization of guidance receptors (B). An unidentified GEF might act in parallel to UNC-73/Trio.

Previous results suggested that UNC-6/Netrin and the receptor UNC-40/DCC can both stimulate and inhibit growth cone protrusion in the same repelled growth cone, which might result in directed protrusion and migration away from UNC-6/Netrin (Norris and Lundquist, 2011). Our results here show that the Rac GEF UNC-73/Trio, the Rac-like GTPases CED-10/Rac and MIG-2/RhoG, and the cytoskeleton-associated molecules UNC-33/CRMP and UNC-44/Ankyrin mediate inhibition of growth cone filopodial protrusion via UNC-5 and UNC-40 Netrin receptors in repulsive axon guidance (Fig. 9). UNC-33 and UNC-44 were required for filopodial inhibition by activated MIG-2 and CED-10, suggesting that they act in a common pathway. UNC-73 is also likely to act in this pathway, as it is a GEF specific for MIG-2 and CED-10 (Wu et al., 2002), and the Rac GEF activity of UNC-73 is abolished by *unc-73(rh40)* (Steven et al., 1998). However, UNC-73 might not be the only GEF that regulates MIG-2 and CED-10 in this process, as *unc-73(rh40)* double mutants with *mig-2* and *ced-10* display increased filopodial protrusion compared with both *unc-73(rh40)* and the *ced-10(n1993); mig-2(mu28)* double mutant. Activated *ced-10* and *mig-2* were epistatic to *unc-73* loss of function (i.e. growth cones in the double mutants displayed inhibited protrusion similar to activated *mig-2* and *ced-10* alone), consistent with the known role of UNC-73/Trio as an upstream Rac regulator.

### UNC-33 is required to inhibit growth cone filopodial protrusion mediated by UNC-6 receptor and Rac signaling

Collapsin response mediator proteins (CRMPs) are required for semaphorin-3A-mediated growth cone collapse through a receptor complex that includes Plexin-A and Neuropilin-1 (Goshima et al., 1995; Takahashi et al., 1999). Here, we demonstrate that UNC-33, a *C. elegans* CRMP-like molecule, is required for the inhibition of growth cone filopodial protrusion caused by the UNC-6/Netrin receptors UNC-40 and UNC-5. In cultured mammalian neurons, CRMP4 (DPYSL3) knockdown results in longer filopodial protrusions and more axon branches on myelin-derived substrates (Alabed et al., 2007), consistent with our results with UNC-33 *in vivo* in *C. elegans*. Rather than acting as a specialized effector of semaphorin signaling, CRMPs might provide a more general mechanism to inhibit growth cone protrusion in response to multiple signals including semaphorins and netrins. Although both growth cone collapse and filopodial inhibition result in reduced growth cone protrusion, it is unclear whether the mechanisms used by CRMPs in these processes are distinct.

*unc-33* and *unc-44* mutations were epistatic to activated MIG-2/RhoG and CED-10/Rac, as the double-mutant growth cones displayed excess filopodial protrusions similar to *unc-33* and *unc-44* mutants alone. This suggests that UNC-33/CRMP and UNC-44/Ankyrin are required for the effects of activated Rac GTPases and that they act downstream of them to mediate filopodial inhibition. CRMPs interact with both the actin and microtubule cytoskeletons. CRMP4 interacts with F-actin *in vitro* (Rosslenbroich et al., 2005), and CRMP1 colocalizes to the actin cytoskeleton of dorsal root ganglion neurons grown in culture (Higurashi et al., 2012). Furthermore, CRMP2 (DPYSL2) physically interacts with tubulin dimers and promotes microtubule assembly (Fukata et al., 2002). Therefore, UNC-33 might directly modulate the actin and/or microtubule cytoskeletons of growth cones in response to UNC-6 to inhibit protrusion.

### Rac signaling and UNC-33 are likely to act downstream of MYR::UNC-40 and MYR::UNC-5

Previous studies suggest that UNC-73/Trio and MIG-2/RhoG act upstream of guidance receptors and affect their localization. Activated MIG-2 caused redistribution of UNC-40::GFP to submembrane structures in axons of the ALM touch neurons (Levy-Strumpf and Culotti, 2007), and UNC-73/Trio acts with the kinesin-like protein VAB-8L to cause increased cell surface localization of the Slit receptor SAX-3/Robo (Watari-Goshima et al., 2007). Furthermore, UNC-33 and UNC-44 affect axon-dendrite trafficking (Maniar et al., 2012). Growth cones were not analyzed in these studies, however. We found that localization of full-length UNC-40::GFP and UNC-5::GFP to VD growth cones was grossly unaffected by *unc-73(rh40)*, *unc-33* and *unc-44*, suggesting that these molecules are not involved in guidance receptor localization to the growth cone in this context. Therefore, our results are consistent with a model in which the Rac-like GTPases MIG-2 and CED-10, UNC-73 and UNC-33 directly regulate cytoskeletal organization downstream of MYR::UNC-40–UNC-5 receptors to inhibit protrusion (Fig. 9). However, it is also possible that these molecules are involved in some aspect of receptor localization, activation or modification in the growth cone that was not detected in our assay.

Expression of full-length UNC-5::GFP resulted in growth cones with reduced protrusion similar to MYR::UNC-40, indicating a gain-of-function effect of transgenic UNC-5::GFP. The loss of UNC-73, UNC-33 and UNC-44 did not affect the localization of UNC-5::GFP to the growth cone nor suppress its inhibitory effect on protrusion, in contrast to the effect of their loss on MYR::UNC-40 and MYR::UNC-5. Possibly, full-length UNC-5::GFP is more effective at inhibiting protrusion than MYR::UNC-40 and MYR::UNC-5. One explanation for this difference is that other pathways might act in parallel to UNC-33 to inhibit protrusion and these might be more effectively engaged by full-length UNC-5::GFP, such that when UNC-33 is absent these pathways can still inhibit protrusion. One such pathway might include the RPM-1/Highwire E3 ubiquitin ligase, the PPM-1/PP2AB phosphatase, and the PHR-binding protein RAE-1, which mediate axon termination in *C. elegans* (Grill et al., 2007, 2012; Tulgren et al., 2011), but is unclear if these act in repulsive Netrin signaling. MYR::UNC-40 and MYR::UNC-5 represent sensitized backgrounds that can be used effectively to probe downstream pathways that might not be evident if full-length molecules were used.

Previous studies suggest that UNC-5 alone mediates repulsion in some contexts (Keleman and Dickson, 2001; Merz et al., 2001). We found that endogenous full-length UNC-5 was required for the effects of both MYR::UNC-40 and MYR::UNC-5, suggesting the involvement of both heterodimeric UNC-40–UNC-5 and homodimeric UNC-5 receptors in growth cone inhibition. That endogenous UNC-5 is required for the effects of MYR::UNC-5 indicates that a MYR::UNC-5 dimer cannot inhibit protrusion. Possibly, a full-length UNC-5 molecule is required for proper trafficking and localization of the activated MYR::UNC-5–UNC-5 receptor, or the extracellular domain of UNC-5 has an UNC-6-independent role in the function of the activated MYR::UNC-5–UNC-5 receptor.

### Rac GTPases have both pro-protrusive and anti-protrusive roles that are regulated by distinct Rac GEFs

The results reported here, combined with previous results (Demarco et al., 2012), show that the UNC-73/Trio and TIAM-1 Rac GEFs have opposite roles in regulating protrusion. MIG-2/RhoG and CED-10/Rac have clear pro-protrusive roles in *C. elegans* neurons (Struckhoff and Lundquist, 2003; Shakir et al., 2008; Demarco et al., 2012), consistent with the effects of Rac in cultured cells (Hall and Nobes, 2000; Hall, 2005; Hall and Lalli, 2010). However, UNC-73 was not required for ectopic protrusions caused by UNC-40 and CDC-42 in the AVM neuron, the axon of which is normally attracted to UNC-6 (Gitai et al., 2003; Demarco et al., 2012). Instead, TIAM-1 was required for the pro-protrusive effects of UNC-40 and CDC-42 in the AVM neuron (Demarco et al., 2012). These results indicate that the Rac-like GTPases MIG-2 and CED-10 are required to both stimulate and inhibit growth cone protrusion, and that distinct GEFs regulate their activities in each role: TIAM-1 to stimulate protrusion and UNC-73 to inhibit protrusion.

### Conclusions

In summary, these results show that UNC-73/Trio, the Rac-like GTPases MIG-2 and CED-10, UNC-44/Ankyrin and UNC-33/CRMP inhibit growth cone filopodial protrusion and are required for inhibition of filopodial protrusion by UNC-6/Netrin receptor signaling. UNC-33 is required to inhibit protrusion by activated MIG-2 and CED-10, suggesting that these molecules act in a common pathway. UNC-73, UNC-33 and UNC-44 are not involved in the accumulation of UNC-40::GFP or UNC-5::GFP to growth cones, suggesting that they might mediate downstream effects of UNC-40 and UNC-5 signaling, possibly on the growth cone cytoskeleton.

## MATERIALS AND METHODS

### Genetic methods

Experiments were performed at 20°C using standard *C. elegans* techniques (Brenner, 1974). Mutations used were: X: *mig-2(mu28)*; I: *unc-73(rh40)*; II: *juIs76* [*Punc-25::gfp*]; IV: *unc-5(e53* and *e152)*, *unc-33(e204* and *e1197)*, *unc-44(e362*, *e1260* and *e1193)*, *ced-10(n1993)*. Chromosomal locations not determined: *lqIs128* [*Punc-25::myr::unc-40::gfp*], *lqIs242 [Punc-25::myr::unc-5::gfp]*, *lqIs204 [Punc-25::ced-10(G12V)]* and *lqIs182* [*Punc-25::mig-2(G16V)*]. Extrachromosomal arrays were attained by injection into the germline, and then integrated into the genome via standard techniques (Mello and Fire, 1995). Multiple (≥3) extrachromosomal transgenic lines of *Punc-25::myr::unc-5::gfp*, *Punc-25::ced-10(G12V)* and *Punc-25::mig-2(G16V)* were analyzed with similar effect, and one was chosen for integration and further analysis. The *mig-2(mu28); ced-10(n1993M+)* strain was balanced with the *nT1* balancer.

The *myr::unc-5::gfp* transgene included the coding region, containing both exons and introns, for the C-terminal 557 residues of the UNC-5A isoform (GenBank accession AAB23867), consisting of all of the cytoplasmic domain but not the transmembrane domain or the extracellular domain. This sequence was placed downstream of the *unc-25* promoter and fused in frame to the myristoylation sequence MGSSKS at the N-terminus as previously described (Gitai et al., 2003) and *gfp* at the C-terminus. The full-length UNC-5::GFP extrachromosomal transgene, *lqEx762*, was generated using the pU5GFP plasmid described previously (Killeen et al., 2002). A plasmid consisting of full-length UNC-40::GFP driven by the *unc-25* promoter in VD/DD neurons was constructed using the *unc-40::gfp* coding region described previously (Levy-Strumpf and Culotti, 2007; Sundararajan and Lundquist, 2012).

### Analysis of axon guidance defects

VD neurons were visualized with a *Punc-25::gfp* transgene, *juIs76* (Jin et al., 1999), which is expressed in all GABAergic neurons, including the 13 VDs. VD axon defects scored include axon guidance (termination before reaching the dorsal nerve cord or wandering at an angle greater than 45° before reaching the dorsal nerve cord) and ectopic branching. Fisher's exact test was used to determine statistical significance between proportions of defective axons.

### Growth cone time-lapse imaging

VD growth cones were imaged as previously described (Norris et al., 2009). Briefly, animals harboring the indicated transgenes were selected 16 h post-hatching at 20°C and placed on a 2% agarose pad with a drop of 10 mM muscimol (Sigma-Aldrich) in M9 (Weinkove et al., 2008), which was allowed to evaporate for 4 min before placing a coverslip over the sample. Growth cones were imaged with a Qimaging Rolera mGi camera on a Leica DMR microscope. Images were acquired at intervals of 120 s, with total duration of time-lapse ranging from 20 to 60 min.

Dynamic projections less than 0.5 µm in width emanating from the growth cone were scored as filopodia. Maximal filopodia length was measured using ImageJ software, and filopodial duration was determined by persistence of the protrusion through time-lapse images. All filopodia on multiple growth cones were analyzed, and at least seven growth cones of each genotype were included in the analysis (at least 25 filopodia). In Fig. 2E, the average length of filopodia was determined from images of growth cones (at least ten growth cones; at least 25 filopodia). The significance of differences was determined by a two-sided *t-*test with unequal variance.

### UNC-5::GFP, UNC-40::GFP and MYR::UNC-40::GFP growth cone analysis

Images of VD growth cones with MYR::UNC-40::GFP and full-length UNC-40::GFP and UNC-5::GFP were taken as described above. Using ImageJ, the perimeters of the growth cones were traced, and the average pixel intensity in the defined growth cone area was reported. At least ten growth cones for each genotype were analyzed, except for *unc-73(rh40); unc-5::gfp*, which were subviable and sterile. *unc-73(rh40); unc-5::gfp* growth cones were not quantified, but those observed showed no gross change in UNC-5::GFP growth cone localization.

## Supplementary Material

Supplementary Material
