# Supplementary material for: The UNC-6/Netrin receptors UNC-40/DCC and UNC-5 inhibit growth cone filopodial protrusion via UNC-73/Trio, Rac-like GTPases and UNC-33/CRMP
Source: Development. 2014 Nov 15;141(22):4395–405. doi: 10.1242/dev.110437 (PMC4302909; doi:10.1242/dev.110437)
Supplement: Supplementary Material [file supp_141_22_4395__index.html]

Supplementary Material 

# The UNC-6/Netrin receptors UNC-40/DCC and UNC-5 inhibit growth cone filopodial protrusion via UNC-73/Trio, Rac-like GTPases and UNC-33/CRMP

## DEV110437 Supplementary Material

**Files in this Data Supplement:**

- Supplementary Material
