# Supplementary material for: The UNC-6/Netrin receptors UNC-40/DCC and UNC-5 inhibit growth cone filopodial protrusion via UNC-73/Trio, Rac-like GTPases and UNC-33/CRMP
Source: Development. 2014 Nov 15;141(22):4395–405. doi: 10.1242/dev.110437 (PMC4302909; doi:10.1242/dev.110437)
Supplement: Supplementary Material [file supp_141.22.4395_DEV110437supp.pdf]

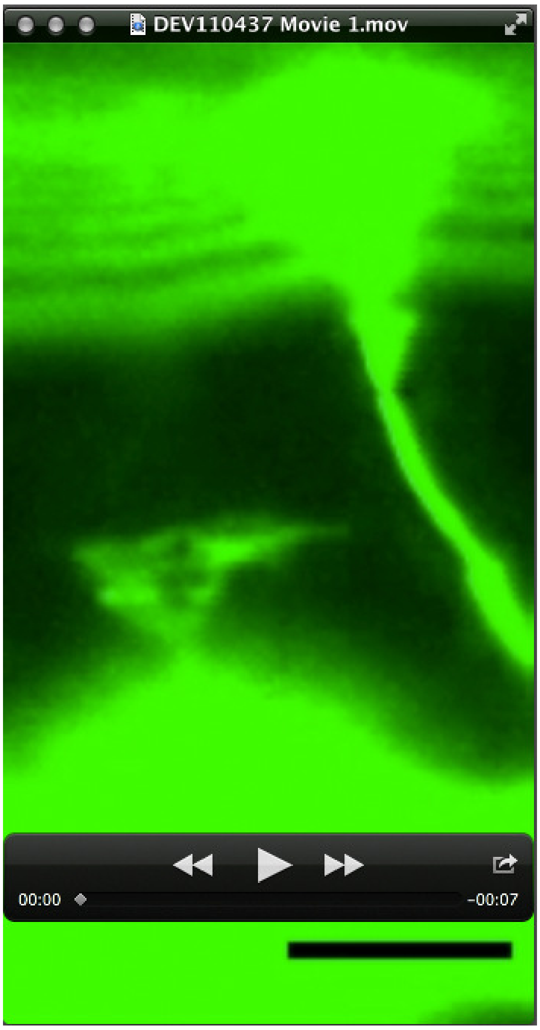

Movie 1. A wild-type VD growth cone.

Time-lapse movie of a wild-type VD growth cone migrating between ventral and dorsal muscle quadrants. Note the dynamic protrusions that extend and retract from the growth cone. Images were captured every 120 s, with a total movie duration of 44 minutes. The scale bar in the first frame represents 5 µm.

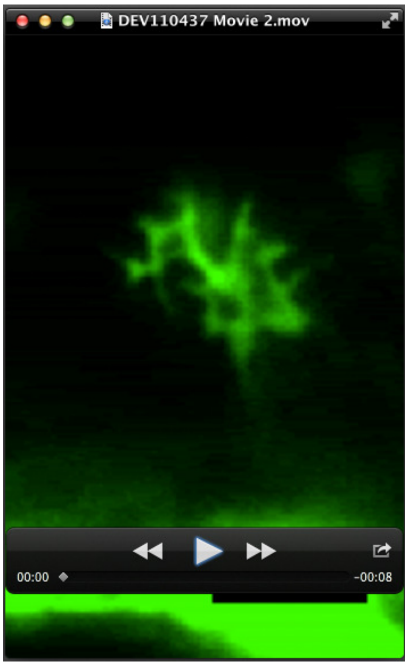

Movie 2. An *unc-5(e53)* mutant VD growth cone.

Time-lapse movie of an *unc-5(e53)* mutant VD growth cone as described in Movie 1. Note the excessively long and persistent protrusions (e.g. on the left side of the growth cone in the second half of the movie). Images were captured every 120 s, with a total movie duration 26 minutes. The scale bar in the first frame represents 5 µm.

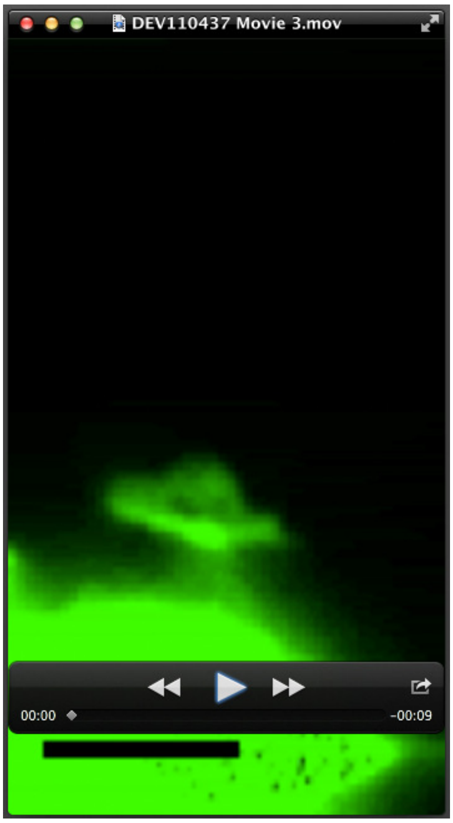

Movie 3. A VD growth cone with *myr::unc-40* VD expression.

Time-lapse movie of a *myr::unc-40* VD growth cone as described in Movie 1. Note the lack of protrusions from the growth cone. Images were captured every 120 s, with a total movie duration of 28 minutes. The scale bar in the first frame represents 5 µm.

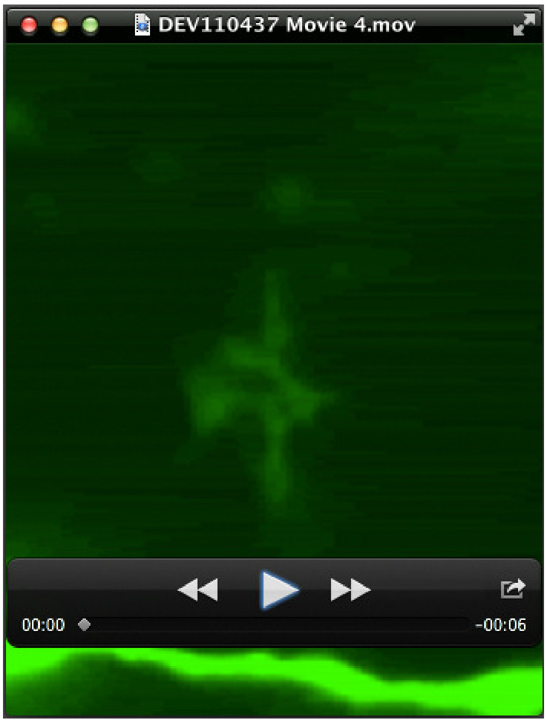

Movie 4. An *unc-73(rh40)* VD growth cone.

Time-lapse movie of an *unc-73(rh40)* VD growth cone as described in Movie 1. Note the excessively long and persistent protrusions from the growth cone. Images were captured every 120 s, with a total movie duration of 26 minutes. The scale bar in the first frame represents 5 µm.

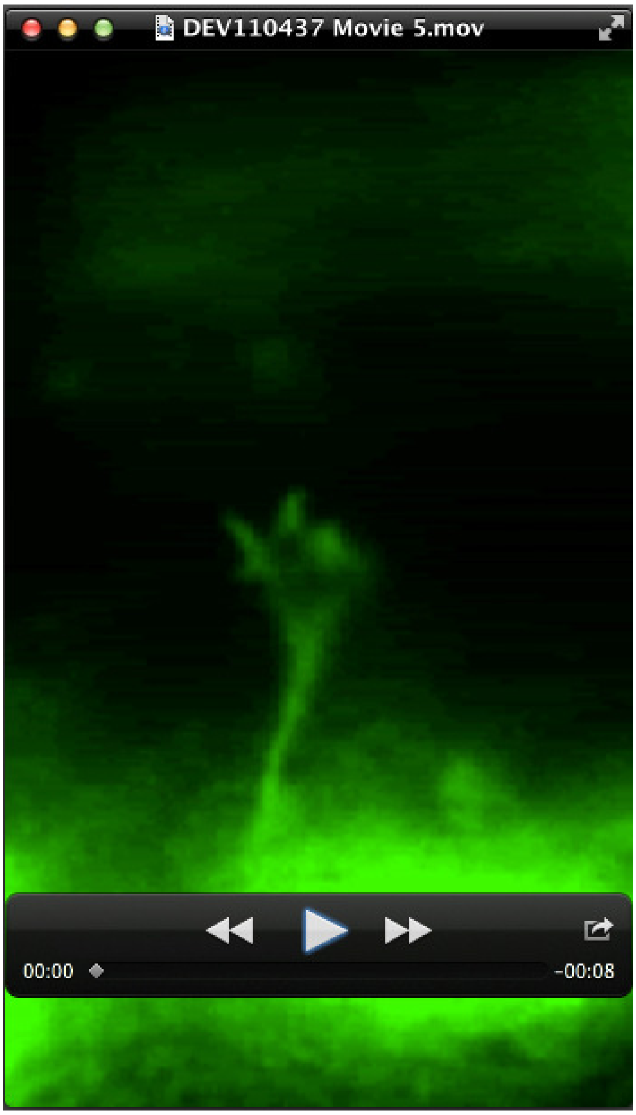

Movie 5. A *ced-10(n1993); mig-2(mu28)* double mutant VD growth cone.
Time-lapse movie of a *ced-10(n1993); mig-2(mu28)* double mutant VD growth cone as described in Movie 1. Note the excessively long protrusions from the dorsal side of the growth cone. Images were captured every 120 s, with a total movie duration of 24 minutes. The scale bar in the first frame represents 5 µm.

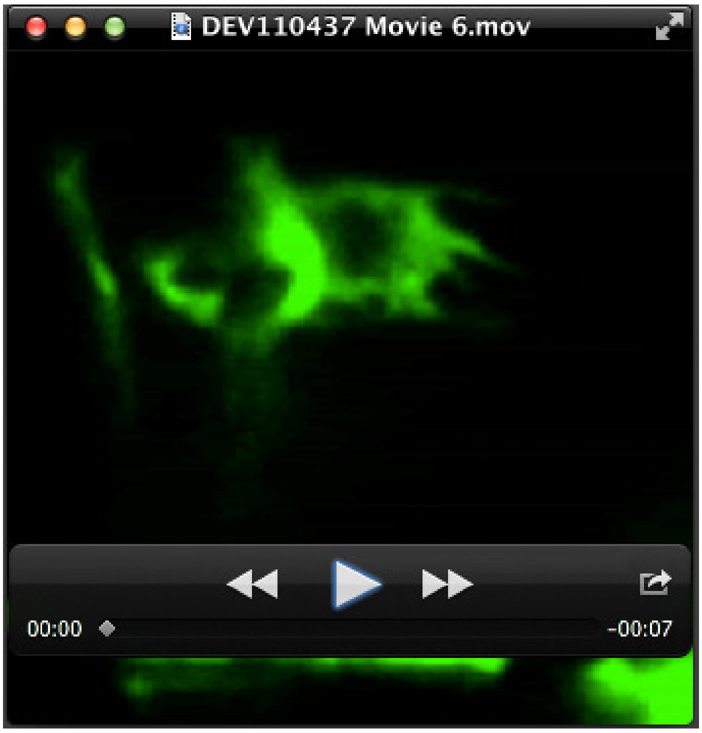

Movie 6. An *unc-33(e204)* mutant VD growth cone.

Time-lapse movie of an *unc-33(e204)* VD growth cone. Note the excessively long and persistent protrusions from the growth cone. Images were captured every 120 s, with a total movie duration of 20 minutes. The scale bar in the first frame of represents 5 μm.

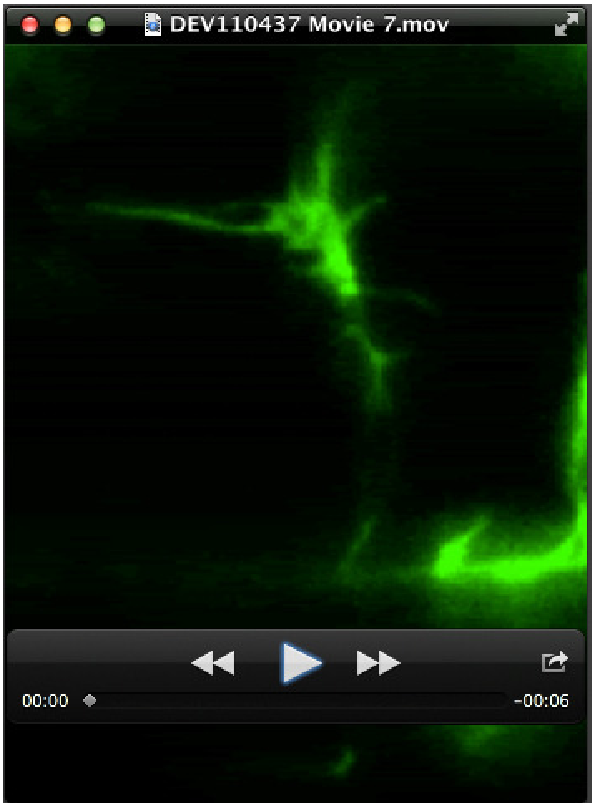

Movie 7. An *unc-44(e362)* mutant VD growth cone.

Time-lapse movie of an *unc-44(e362)* VD growth cone. Note the excessively long and persistent protrusions from the growth cone. Images were captured every 120 s, with a total movie duration of 20 minutes. The scale bar in the first frame represents 10 µm.

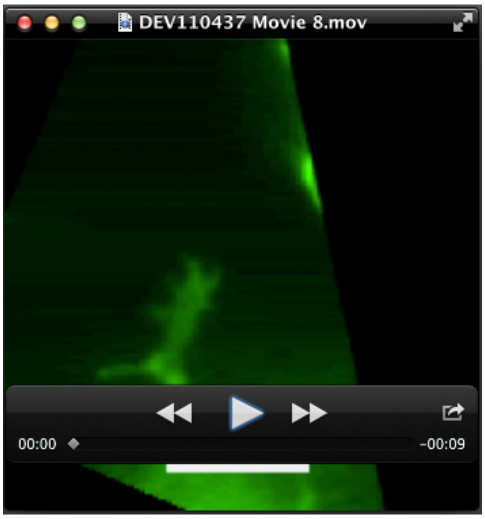

Movie 8. An *unc-73(rh40)* mutant growth cone with *myr::unc-40* expression. Time-lapse movie of a *myr::unc-40; unc-73(rh40)* VD growth cone. Note the excessively long and persistent protrusions from the growth cone. Images were captured every 120s, with a total movie duration of 28 minutes. The scale bar in the first frame represents 5 µm.

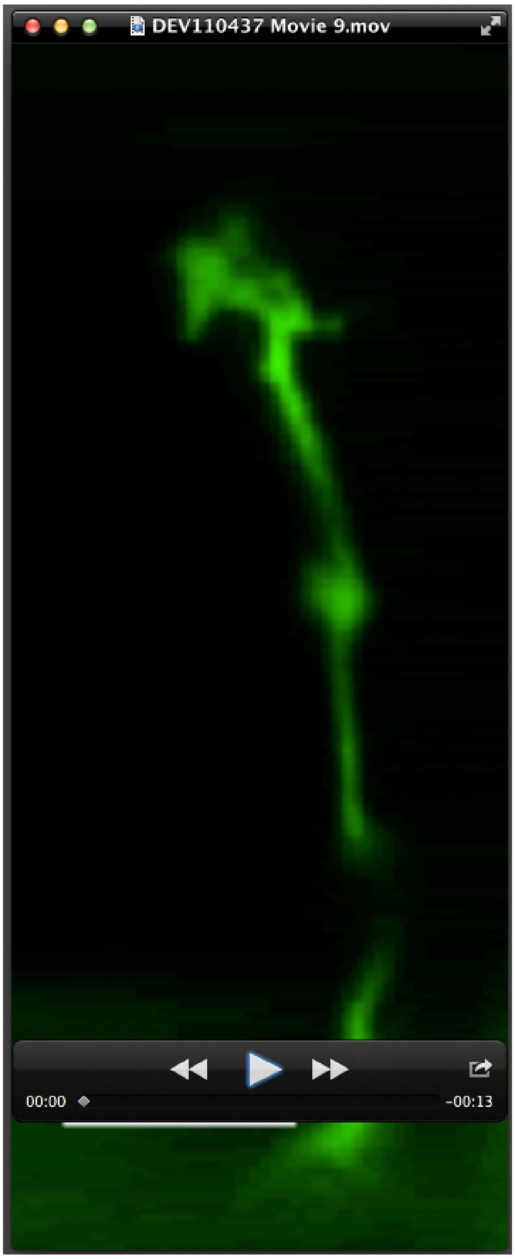

Movie 9. A VD growth cone expressing activated *mig-2(G16V).*

Time-lapse movie of a VD growth cone with expression of activated *mig-2(G12V)* as described in Movie 1. Note the reduction in protrusions from the growth cone. Images were captured every 120s, with a total movie duration of 40 minutes. The scale bar in the first frame represents 5 µm.

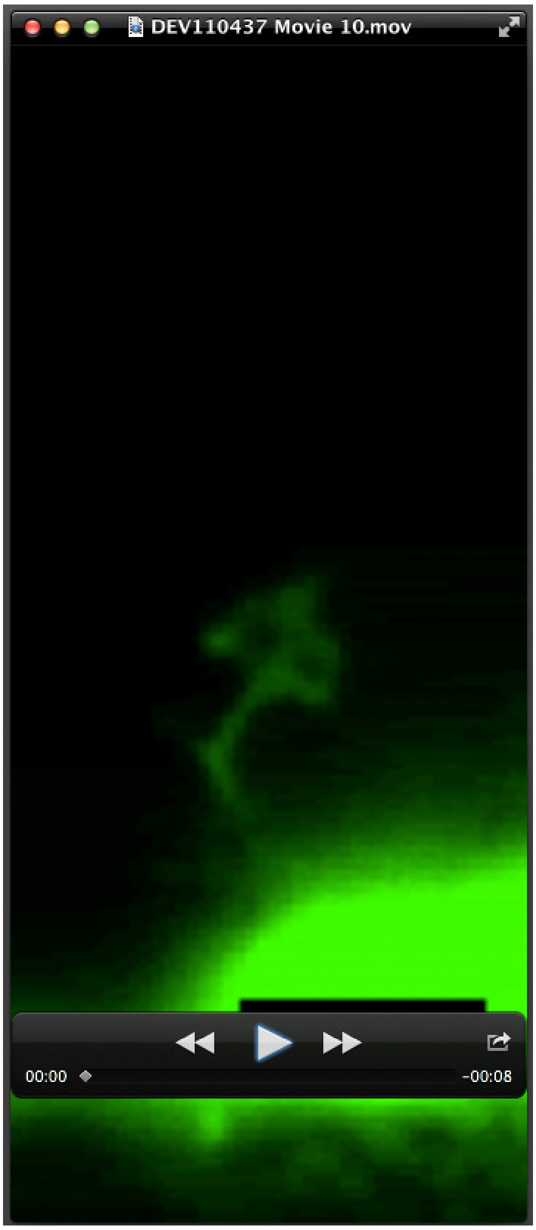

Movie 10. An *unc-73(rh40)* mutant growth cone with activated *mig-2(G16V)* expression.

Time-lapse movie of an *unc-73(rh40)* mutant growth cone with *mig-2(G16V)* expression. Note the reduction in protrusions from the growth cone. Images were captured every 120s, with a total movie duration of 24 minutes. The scale bar in the first frame represents 5 µm.

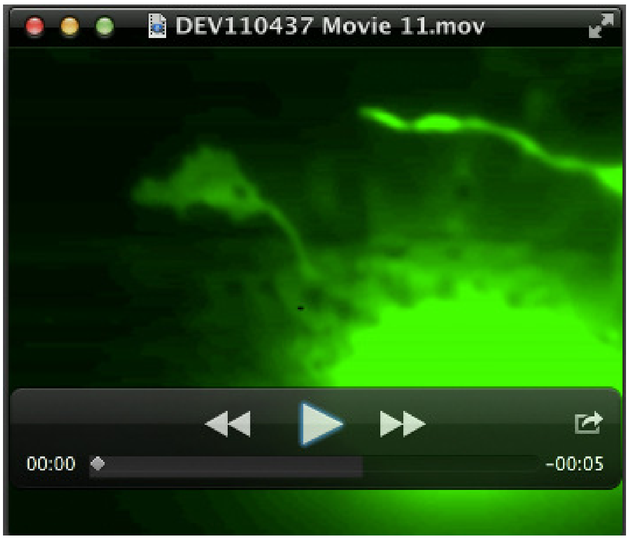

Movie 11. A VD growth cone with *myr::unc-5* expression.

Time-lapse movie of a *myr::unc-5* VD growth cone. Note the reduction in protrusions from the growth cone. Images were captured every 120s, with a total movie duration of 34 minutes. The scale bar in the first frame represents 10 µm.
